# Supplementary figures and images for: Invasion and high-elevation acclimation of the red imported fire ant, Solenopsis invicta, in the southern Blue Ridge Escarpment region of North America
Source: PLoS One. 2020 May 1;15(5):e0232264. doi: 10.1371/journal.pone.0232264 (PMC7194361; doi:10.1371/journal.pone.0232264)

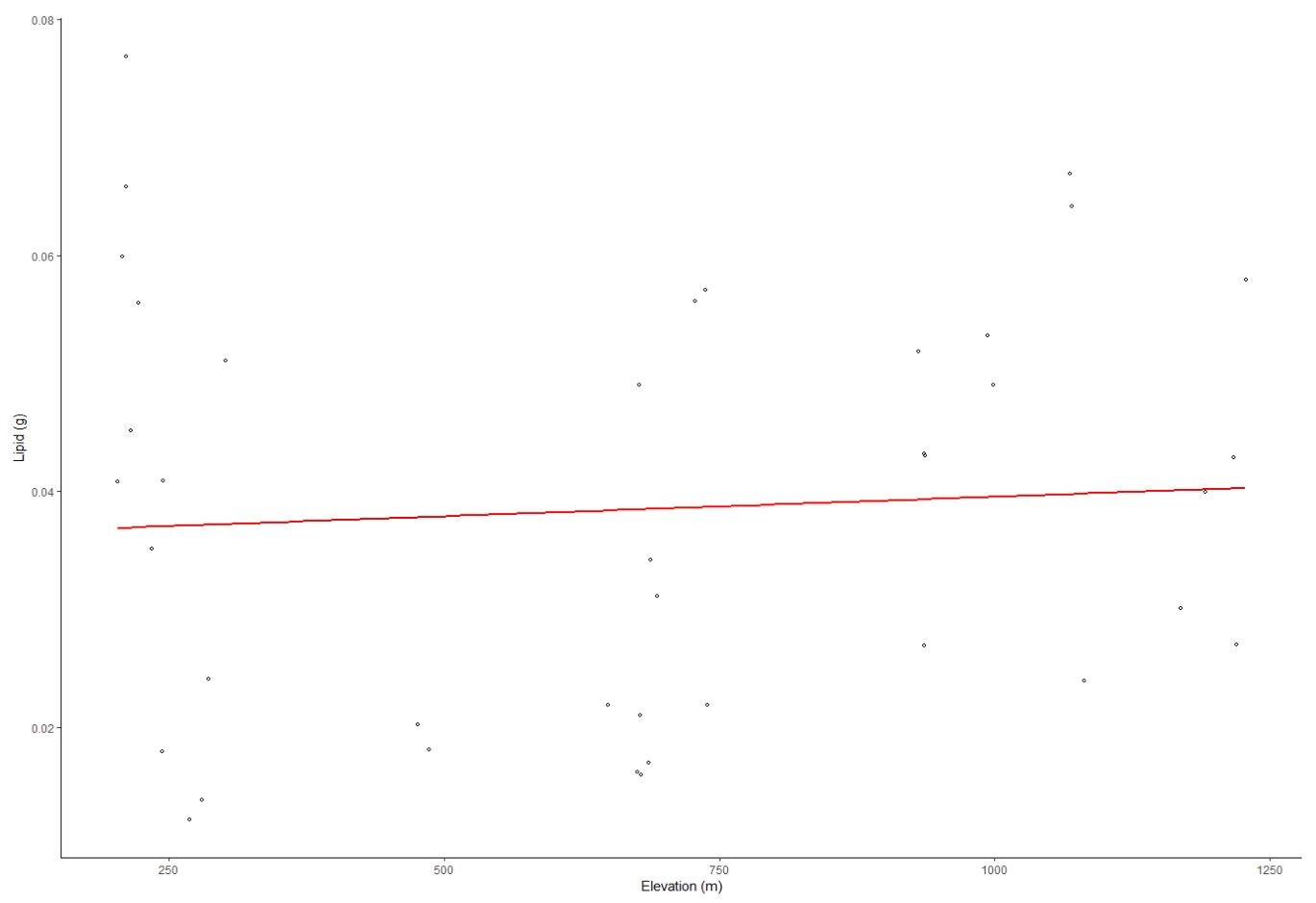

Supplement: S1 Fig — (TIF) [file pone.0232264.s001.tif]
